# Relevant Journals for Identifying Implementation Science Articles: Results of an International Implementation Science Expert Survey

**DOI:** 10.3389/fpubh.2021.639192

**Published:** 2021-04-30

**Authors:** Juliane Mielke, Thekla Brunkert, Leah L. Zullig, Hayden B. Bosworth, Mieke Deschodt, Michael Simon, Sabina De Geest

**Affiliations:** ^1^Department Public Health, Institute of Nursing Science, University of Basel, Basel, Switzerland; ^2^Manitoba Centre for Health Policy, Department of Community Health Sciences, Rady Faculty of Health Sciences, University of Manitoba, Winnipeg, MB, Canada; ^3^Center for Innovation to Accelerate Discovery and Practice Transformation, Durham Veterans Affairs Health Care and System, Department of Population Health Sciences, School of Medicine, Duke University, Durham, NC, United States; ^4^Division of General Internal Medicine, Department of Medicine, Duke University School of Medicine, Durham, NC, United States; ^5^School of Nursing, Duke University, Durham, NC, United States; ^6^Department of Health Policy and Management, Gillings School of Global Public Health, University of North Carolina at Chapel Hill, Chapel Hill, NC, United States; ^7^Department of Public Health and Primary Care, Gerontology, and Geriatrics, KU Leuven, Leuven, Belgium; ^8^Healthcare and Ethics, Faculty of Medicine and Life Sciences, UHasselt, Hasselt, Belgium; ^9^Department of Public Health and Primary Care, Academic Center for Nursing and Midwifery, KU Leuven, Leuven, Belgium

**Keywords:** implementation science, implementation science journals, survey, literature review, translational research, dissemination

## Abstract

In implementation science (IS), conducting well-targeted and reproducible literature searches is challenging due to non-specific and varying terminology that is fragmented over multiple disciplines. A list of journals that publish IS-relevant content for use in search strings can support this process. We conducted a cross-sectional online survey of 56 Australian, European, and North American IS experts to identify and prioritize relevant journals that publish IS articles. Journals' relevance was assessed by providing each with a list of 12 journals, to which they were encouraged to add additional journal names and comments as free text. We also assessed which journals had published special IS-focused issues—identified *via* PubMed and Google searches—over the last 20 years. Data were analyzed descriptively. Between February 28 and March 15, 2020, a purposive sample of 34/56 experts participated in the survey (response rate: 60.7%). *Implementation Science* and *BMC Health Services Research* were perceived as relevant by 97.1% of participants; other journals' relevance varied internationally. Experts proposed 50 additional journals from various clinical fields and health science disciplines. We identified 12 calls and 53 special issues on IS published within various journals and research fields. Experts' comments confirmed the described challenges in identifying IS literature. This report presents experts' ratings of IS journals, which can be included in strategies supporting searches of IS evidence. However, challenges in identifying IS evidence remain geographically and interdisciplinary. Further investment is needed to develop reproducible search strings to capture IS evidence as an important step in improving IS research quality.

## Introduction

Bridging the gap between research and practice using scientific methods is the central aim of implementation science (IS) which can be defined as the “scientific study of methods to promote the systematic uptake of research findings and other evidence-based practices into routine practice, and, hence, to improve the quality and effectiveness of health services and care” (1). IS studies typically require an expansive cross-disciplinary understanding of relevant empirical findings and of whether and where they have been implemented. To ensure that research is novel, necessary, and attentive to existing work, each research project should begin with a search of relevant IS literature (2). However, this search process is hampered by a lack of unified definitions and conceptualizations, as well as by suboptimal indexing: a plethora of terms are used across disciplines, varying over time and geography (e.g., dissemination and implementation science, knowledge translation, research utilization) (3–5). This lack of consistency applies not only to terminology, but also to the definition of IS itself (6). In addition, some aspects of the IS methodology overlap with methods from other fields, all of which have their own specific language and labeling (e.g., improvement science) (7, 8). This results in heterogeneity and inconsistencies in operationalization challenge the development of precise search strings, thereby impacting the identification of relevant literature (9).

Problems with the sensitivity and specificity of systematic IS literature searches were already being reported in 2010 (10, 11). In response, Lokker et al. and McKibbon et al. developed search filters to identify different types of IS articles (general, theoretical, IS instruments, application-focused) from CINAHL and MEDLINE (10, 11). For MEDLINE, these filters' sensitivity ranged from 85 to 90%, with specificity ranging from 65 to 75% depending on the type of article (11). For CINAHL, their retrieval efficacy was comparable, i.e., they resulted in a large number of results, many of which were irrelevant (10). In contrast, search strings for clear, well-defined concepts, such as *randomized clinical trials* (RCTs), showed both sensitivity and specificity over 99%. Concepts with a high variability of search terms such as patient and public involvement reach comparable retrieval rates as IS search strings (12–14).

Challenges in developing precise IS search strings are also described in other systematic reviews (15, 16) and similar to our own experience in an ongoing mapping review project, the ImplemeNtation science State of research ProjECT (INSPECT) (17). INSPECT involves a group of experts in nursing, health services research, implementation science, public health and health policy who guided the formation of an extensive search string intended to capture the existing status of IS as a scientific discipline. In contrast to previous work, this INSPECT concerns the total IS literature identified through our search string. However, similar to prior reviews, the INSPECT project is affected by limited sensitivity and specificity which challenges the identification of relevant IS literature.

Over the past two decades, IS has gained increasing importance in various health related disciplines and other fields (e.g., environmental sciences) (18). This importance is reflected in the expanding number of journals not only addressing IS specifically but publishing special issues to showcase IS studies and methodological issues in IS in their respective fields. In a field as broad and rapidly evolving as IS, a growing number of empirical and theoretical IS papers are scattered over diverse peer-reviewed journals (19). In combination with the indistinct terminology identification of relevant evidence is even more challenging.

In 2019, the National Library of Medicine introduced “implementation science” as a medical subject heading (MeSH) in PubMed. This will aid literature searches considerably and should eventually decrease the challenge of finding IS-related articles in the future.

In order to access relevant IS literature published before 2019 (and probably also after until some congruence in labeling is adopted internationally), a more targeted approach is needed. One pragmatic step in this targeted direction is to compile a list of relevant journals for IS search strategies, which will help to narrow the search. Further, as studies in various fields have demonstrated that articles published in special issues are often published more quickly and with higher impact (citation rate per article) than regular articles, (20, 21) these special issues might be particularly useful to help identify relevant evidence.

Therefore, our primary objective was to identify and prioritize journals that publish IS articles with the goal of summarizing current journals where IS research may be located from an IS expert viewpoint. We further assessed which journals have published special issues about IS over the last 20 years.

## Methods

### Design, Setting, Sample

We developed and administered a cross-sectional online survey targeting international IS experts and invited a purposive sample of 56 from Australia, Europe, and North America to participate. To achieve a high level of expertise, the sampling pool was composed of IS practitioners and researchers, we identified from the collaboration networks published by Norton et al. (22), and on the website of the European Implementation Collaborative. Since implementation scientists are disproportionately concentrated in the US and Europe, we included experts with guidance from the articles' authors, to ensure international geographic representation and balance the sample to the extent possible. While there are many complementary disciplines, e.g., improvement science, our research objective focused specifically on identifying IS literature. Therefore, we engaged experts working specifically in IS. The reporting of this study adhered to the STROBE Statement as well as the Checklist for Reporting Results of Internet E-surveys (CHERRIES) (23, 24).

### Variables and Measurement

First, we assessed the perceived relevance of journals identified in a literature search for INSPECT (17). An extensive search string was developed, using text words and MeSH terms referring to IS (Table 1). Almost 11,000 hits (*N* = 10,904) were identified, published in 2,461 various journals. We selected the 12 journals most commonly identified (more than 60 times), which represent 31.4% of all hits. Experts could rate the relevance of each journal on a 4-point Likert scale. Perception responses ranged from 1 (“not at all”) to 4 (“definitely”), with 5 signifying “journal not known.” Perception scores were dichotomized as either “relevant” (ratings of 3 or 4) or “not relevant” (ratings of 1 or 2); ratings of 5 were set as missing. Next, experts were invited to indicate any other journals they deemed important for the identification of IS articles. Finally, demographic characteristics including country of residence, field of research, and years of experience in implementation research were gathered.

**Table 1:** Search string INSPECT project in PubMed until 31.12.2019 achieved 10,904 hits.

Diffusion of innovation*[Title/Abstract] OR dissemination science[Title/Abstract] OR Implementation research[Title/Abstract] OR Implementation science[Title/Abstract] OR “implementation science is”[Journal] OR Improvement science[Title/Abstract] OR Knowledge to action[Title/Abstract] OR Know-do gap[Title/Abstract] OR Knowledge transfer[Title/Abstract] OR knowledge translation[Title/Abstract] OR Knowledge utilization[Title/Abstract] OR Research implementation[Title/Abstract] OR Research utilization[Title/Abstract] OR “translational behavioral medicine”[Journal] OR Translational science[Title/Abstract]

### Data Collection

Data were collected between February 28 and March 15, 2020. A closed survey was developed using the online https://www.umfrageonline.com/ software and its usability and technical functionality was pilot-tested by this report's three authors (LL, TB, SDG). A personalized letter (English) describing the study and providing a survey link was emailed to the experts. To prevent entries to the survey for a second time, the online software tool used cookies and IP addresses. After 1 week, a reminder was sent to all IS experts because the survey was de-identified.

Participation in the online survey was entirely voluntary, with consent implied by answering and returning the survey. Data were fully anonymized. Following Swiss ethical standards, Art. 2, Federal Act on Research involving Human Beings (Human Research Act, HRA), we neither required nor requested ethical approval.

### Data Analysis

The anonymized data were analyzed descriptively using IBM® SPSS® 25.0.0. Means and standard deviations (SDs) were reported for normally distributed, and medians and interquartile ranges (IQR) for non-normally distributed data. Expert comments were analyzed using content analysis (25, 26). Categories were created based on inductive approach.

### Web-based Search for Implementation Science Special Issues

*Via* PubMed and Google, we searched for IS special issues using the search terms “special issue” AND “implementation science.” All special issues related to IS in healthcare from 2000 until March 2020 were included. Additionally, we manually searched all journals listed in our survey and suggested by IS experts. Information on journal name, special issue title, volume (issue), publication or submission date, number of papers included in the special issue, the journal's impact factor and *h*-index, and country were extracted and presented in a table.

## Results

### Implementation Science Experts' Characteristics

Of the 56 invited IS experts, 34 experts from 12 countries, participated in the study, corresponding to a response rate of 60.7% (Supplementary Figure 1). Their fields of professional activity included public health (*n* = 9; 26.5%), social science (*n* = 6; 17.6%), mental health (*n* = 4; 11.8%), acute care (*n* = 2; 5.9%), psychology (*n* = 2; 5.9%), primary care (*n* = 1; 2.9%), social work (*n* = 1; 2.9%), or other (*n* = 9; 26.5%). They had a median of 12 years' experience in implementation research (IQR = 12.8; range 4–30).

### Perceived Relevance of Journals

**Figure 1:**
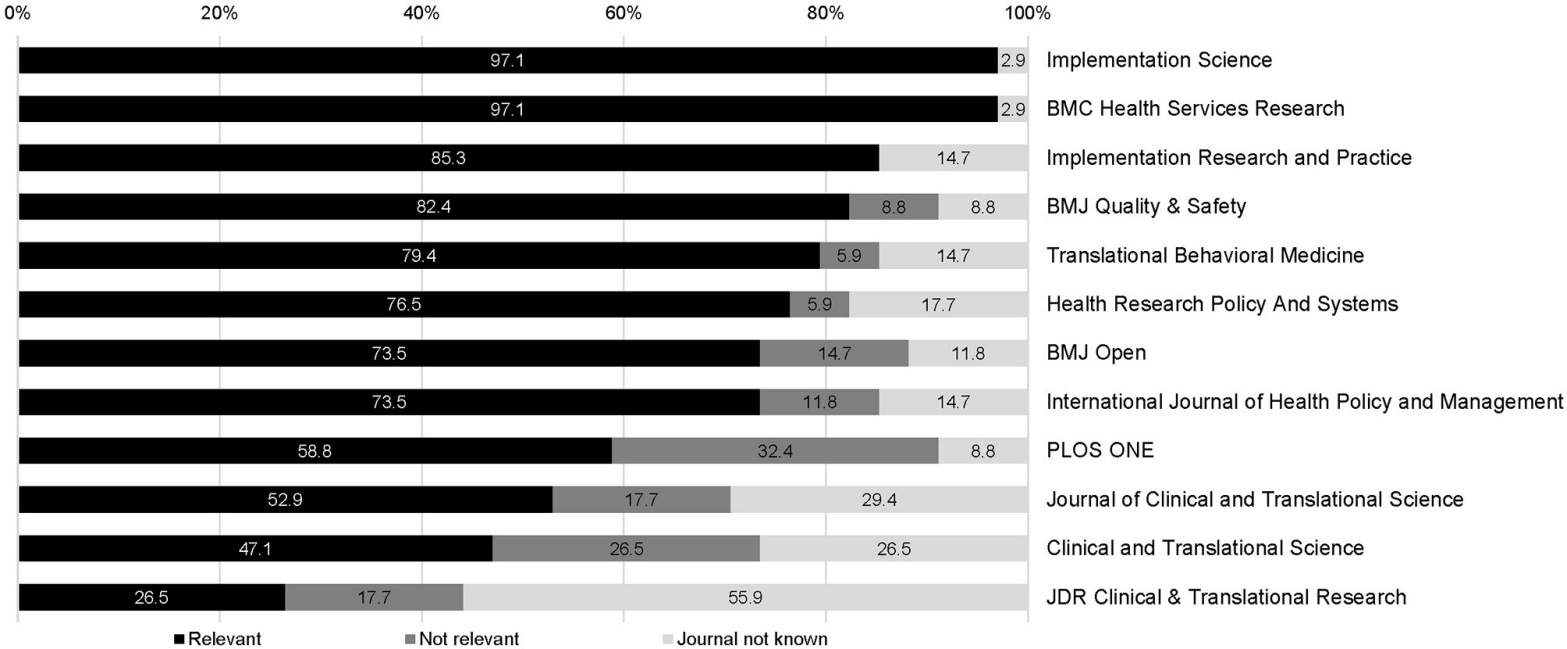
Perceived relevance^a^ of IS journals (in %) to identify IS articles in total (*N* = 34). ^a^Dichotomized as “relevant” (“definitely” or “somewhat”) and “not relevant” (“very little” or “not at all”).

**Figure 2:**
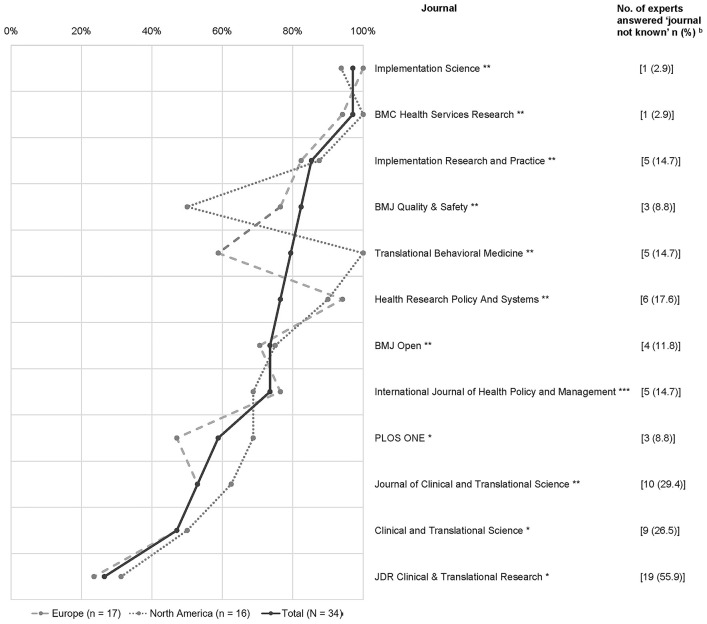
International variation in perceived relevance^a^ of journals by European and North American experts. ^a^Dichotomized as “relevant” (“definitely” or “somewhat”); ^b^answer option “journal not known” set as missing when calculating percentages Country of publication: *USA, **Europe, ***Iran.

The perceived relevance of the listed journals regarding IS article identification ranged from 26.5 to 97.1% (Figure 1). Overall, *Implementation Science* and *BMC* (BioMed Central) *Health Services Research* were perceived as relevant by 33 experts (97.1%), followed by *Implementation Research and Practice* (*n* = 29; 85.3%) and *BMJ* (British Medical Journal) *Quality and Safety* (*n* = 28; 82.4%). Two journals received relevance ratings below 50%: *Clinical and Translational Science* and *JDR* (Journal of Dental Research) *Clinical and Translational Research*, which was unknown to over half (55.9%) of the participants. Also, European and North American experts' perceptions varied strongly regarding three other journals: *BMJ Quality and Safety* (respectively, 76.5 vs. 50%), *Translational Behavioral Medicine* (respectively, 58.8 vs. 100%), and *PLoS ONE* (respectively, 47.1 vs. 68.8%) (Figure 2).

### Other Relevant Journals

Forty-seven other relevant journals publishing IS articles were suggested by 31 experts, referring to various fields of research (e.g., public health, mental health, or psychology) (Table 2). Most suggested journals oriented primarily toward clinicians in a particular field, such as internal medicine or mental health, but that publish implementation-relevant work. These journals included, for example, the *American Journal of Public Health*, the *Journal of General Internal Medicine*, and *Psychiatric Services*.

**Table 2:** Other journals experts deemed important in view of identification of IS articles (*n* = 31).

**Journal names**	***n* (%)**
**Journals that were denoted more than once each**	
Implementation Science Communications	16 (51.6)
Administration and Policy in Mental Health and Mental Health Services Research	12 (38.7)
American Journal of Public Health (AJPH)	5 (13.5)
Journal General Internal Medicine	3 (9.7)
Prevention Science	3 (9.7)
Evidence and Policy	3 (9.7)
Psychiatric Services	3 (9.7)
Journal of Medical Internet Research (JMIR)	2 (6.5)
Journal of the American Medical Association (JAMA)	2 (6.5)
American Journal of Preventive Medicine	2 (6.5)
Frontiers in Public Health	2 (6.5)
JAMA Internal Medicine	2 (6.5)
Journal of Community Psychology	2 (6.5)
Milbank Quarterly	2 (6.5)
World Views on Evidence Based Nursing	2 (6.5)
**Journals that were denoted once each**	
BMC Public Health	
American Journal of Community Psychology	
BMC Globalization and Health	
BMC Medical Education	
BMJ	
Cancer	
Clinical Psychology: Science and Practice	
Community Mental Health Journal	
Ethnicity and Disease	
Health Affairs	
Health Behavior Research	
Health Services Research	
International Journal for Equity in Health	
International Journal of Mental Health Systems	
JAMA Dermatology	
JAMA Oncology	
JMIR Formative Research	
Joint Commission Journal on Quality and Patient Safety	
Journal of Acquired Immune Deficiency Syndromes (JAIDS)	
Journal of Clinical Child and Adolescent Psychology	
Journal of Evidence-Based Social Work	
Journal of Evidence-Informed Social Work	
Journal of Health Services Research and Policy	
Medical Care	
Palgrave Communications	
Pilot and Feasibility Studies	
Psychological Services	
Research on Social Work Practice	
Social Science and Medicine	
Stanford Social Innovation Review	
The Gerontologist	
The Journal of Behavioral Health Services and Research	

### Comments of Experts

Using a free-text comment box, 12 experts provided comments and confirmed the described challenges in identifying IS literature. Using content analysis, we developed three categories: (1) A plethora of terms used for IS: “IS articles are highly variable […] depending on how one interprets IS (even within the context of the Mittman and Eccles definition) […] It is really soiled, and even articles that appear as IS are sometimes (or even often) not really IS (i.e., way outside the conceptualization of IS, such as only focusing on implementing something vs. studying the implementation of it)” (#3; Other). (2) Methodological overlap of IS with other fields of research and scattering of IS evidence across disciplines: “There are hundreds [refers to journals; author's note] as in my experience implementation-relevant work is now being published in almost every field […]. IS is very much an integrative field” (#1; Other). (3) Individual considerations to access relevant IS literature: “So for me it depends on the field of research: for my area, I would add the specific journals that I know where such research is published, although it might be only 2-3 articles per year” (#10; Other).

### Journals With Special Calls for Implementation Science

We identified 12 calls for ongoing IS special issues with papers to be submitted from May 2020 to January 2021 (Supplementary Table 1), as well as 53 others published between 2000 and 2020 (Supplementary Table 2). These special issues are linked to 49 journals from various fields of research. Nine journals have published two or more IS-focused special issues: *Administration and Policy in Mental Health and Mental Health Services Research* (*n* = 4); *American Journal of Preventive Medicine* (*n* = 3); *Clinical Psychology: Science and Practice* (*n* = 2); *Frontiers in Public Health* (*n* = 4); *Health Psychology* (*n* = 2); the *International Journal of Environmental Research and Public Health* (*n* = 4); the *Journal of Clinical Child and Adolescent Psychology* (*n* = 2); the *Journal of Community Psychology* (*n* = 2); *Nursing Research and Practice* (*n* = 2). The geographical location of these journals is Europe (*n* = 24), US (*n* = 23) and Africa (*n* = 2). They publish in open access (*n* = 11), hybrid open access (*n* = 31) or non-open access (*n* = 7).

## Discussion

Accessing and synthesizing available evidence is an essential first step in research and required to successfully bridge the gap between research and real-world settings. However, challenges to the identification of available IS evidence were already being reported a decade ago and continue to cause avoidable research waste (2, 8–11, 27). To ensure effective retrieval and reproducibility of searches, a search strategy should entail all relevant search terms for a concept to be studied—both text words and MeSH terms—that can be combined using Boolean operators. Further validated filters can be applied to support finer targeting. Previous studies about IS search filters provide an overview of relevant terms to build up search strings to identify various types of implementation research (10, 11). In addition, the recent introduction of “implementation science” as a MeSH term for PubMed searches will certainly support researchers' literature searches.

Still, the challenge of conceptual inconsistency remains. To cope with this inconsistency, journals can be included in search strings to supplement searches using text words and MeSH terms. Our cross-sectional online survey of international IS experts provides a basic selection of such journals.

Journals identified *via* the work reported here correspond partly to the findings of Norton et al. (22), providing an overview of the 20 journals in which researchers focusing on dissemination and implementation most frequently found IS articles. Of Norton et al.'s 20 journals, 13 were also considered relevant by this study's experts (22). Inter-study differences in those journals' perceived relevance may be due to sampling differences: 73.6% of Norton et al.'s participants were from the US. Our study shows that some studies' perceived relevance depends on the experts' geographical location, which might be related to geographic differences in IS operationalization.

Another challenge in identifying IS-relevant evidence is the heterogeneity and fragmentation of IS across research fields and disciplines which is highlighted by the variation of journals identified in our survey (4, 6, 10, 11). Our experts also noted and reflected on this, as evidenced by their comments and journal recommendations. As IS is inherently multidisciplinary, articles can be published in diverse journals and databases. This is reflected in the wide and growing range of journals publishing special IS issues. This heterogeneity and rapidly increasing complexity are not only major challenges to IS researchers, but indicators of the barriers other clinical researchers also encounter daily in their fields. And if these challenges impede researchers' access to effective evidence, then the first crucial step of research—identification of that evidence—is impossible.

This work's most notable strength is its inclusion of an international expert panel. We had a high response rate. However, our sampling approach might have resulted in underrepresentation of IS experts from Canada or Australia and underrepresentation from some regions (e.g., Africa, Asia, or South America) as we were not able to identify IS experts in the latter continents. Our difficulty to identify experts in certain parts of the world might point to the major potential for IS activities in those areas (28, 29). Further, we carefully selected our 12 pre-defined journals based on a prior systematic literature search. However, as mentioned by our experts, the survey list focused almost exclusively on IS-specific journals, excluding subject-specific journals, which are also publishing increasingly articles on IS. To maintain a flexible perspective, we provided a free text box to add further journals perceived as relevant by the experts. But no major additions appeared, despite the journals Implementation Science Communications and Administration and Policy in Mental Health and Mental Health Services Research. By that, our search strategy provides a very pragmatic approach to assessing relevant IS evidence effectively. To ensure a more comprehensive identification of specific subject related IS studies, further journals might be added to our list. In that regard, a network analysis might be an objective way forward to evaluate journal‘s influences and relationships. Network analysis requires a subset of all possible IS-related journals. However, identification of all relevant journals through a literature search in a field as scattered and as fast evolving than IS would be prohibitive. Therefore, our list of ranked journals might inform network analysis about IS journals as already published in the field of information systems (30). In accordance with the regulations of the University Ethics Committee, our survey was anonymized. Therefore, we were not able to account for non-response bias, i.e., to assess how respondents vary from non-respondents, which might potentially bias the results.

Since IS rapidly develops, terminology evolves and further journals will arise. Our approach to access relevant IS evidence should be further developed or alternative approaches considered. Testing those approaches against each other will help to quantify differences in effectivity. As subjectively derived search strings (expert based) are often prone to methodological criticism, the development of objectively derived search strategies (research based) could be an alternative approach to identify IS journals (31). This approach entails a four-step process: first, a subset of IS journals is generated, of which a search strategy is developed in a second step to identify this subset journals. Third, the developed search strategy is validated against a validation set containing different journals and finally the process is documented (31). However, key for developing empirical search strings is the availability of papers relevant to the studied topic in order to achieve sufficient sensitivity (close to 90%).

## Conclusion

Overall, based on expert ratings, this study illustrates the perceived relevance of journals publishing IS-relevant articles. We found considerable international variability in these journals' relevance ratings. Considering literature searches' importance to the research process, this information will simplify and accelerate the development of reproducible searches for IS articles.

However, variations in terminology and conceptualization cause inconsistency in interregional and interdisciplinary research; challenges in identifying and reviewing IS evidence from outside the most accessible sources remain. Investing more time to develop reproducible search strings to capture IS evidence would be an important step in improving IS research quality.

## Data Availability Statement

The raw data supporting the conclusions of this article will be made available by the authors, without undue reservation.

## Ethics Statement

Ethical review and approval was not required for the study on human participants in accordance with the local legislation and institutional requirements. The patients/participants provided their written informed consent to participate in this study.

## Author Contributions

JM, LZ, SD, and TB conceived and designed the study and conducted the data collection. JM performed data analysis and drafted the manuscript. HB, LZ, MD, SD, TB, and MS contributed to the analysis and interpretation of data as well as to drafting the manuscript. All authors read and approved the final manuscript.

## Conflict of Interest

HB reports research grants from the PhRMA Foundation, Proteus Digital Health, Otsuka, Novo Nordisk, Sanofi, and Improved Patient Outcomes, as well as consulting from Sanofi, Novartis, Otsuka, Abbott, Preventric Diagnostics, and the Medicines Company. LZ reports research support from Sanofi, Proteus Digital Health, and the PhRMA Foundation, as well as consulting for Novartis. SD consults for Sanofi. The remaining authors declare that the research was conducted in the absence of any commercial or financial relationships that could be construed as a potential conflict of interest.

## Abbreviations

BMC: BioMed Central
BMJ: British Medical Journal
INSPECT: ImplemeNtation science State of research ProjECT
IS: implementation science
IQR: interquartile range
JDR: Journal of Dental Research
MeSH: medical subject heading
RCT: randomized controlled trial.
