# Supplementary material for: Relevant Journals for Identifying Implementation Science Articles: Results of an International Implementation Science Expert Survey
Source: Front Public Health. 2021 Apr 30;9:639192. doi: 10.3389/fpubh.2021.639192 (PMC8119993; doi:10.3389/fpubh.2021.639192)
Supplement: Supplementary file 1 [file Table_1.DOCX]

Supplementary Material

1. **Supplementary Table S1.** Overview of calls for implementation science special issues (from year 2000 to January 2021)

| **Journal name** | **Title special issue** | **To be submitted** | **IF ^a^ / *h*-index ^b^** | **Country** |
| --- | --- | --- | --- | --- |
| Pilot and Feasibility Studies * | Implementation science and practice: pilot and feasibility studies from the field | 2020 | 1.760 / 12 | UK |
| Journal of Alternative and Complementary Medicine * | Effectiveness, Implementation and Dissemination Research in Integrative Health | May 2020 | 1.868 / 80 | US |
| International Journal of Environmental Research and Public Health | Closing the Implementation Gap in Reproductive, Maternal, Newborn and Child Health in Low- and Middle-Income Countries | June 2020 | 2.620 / 78 | CH |
| Journal of Clinical Medicine Section Oncology * | Cancer Rehabilitation and Survivorship | Jul 2020 | 5.688 / 16 | CH |
| Journal of Health Organization and Management ** | Implementation Science in Health Care Organization, Management and Policy | Aug 2020 | 1.470 / 35 | UK |
| Journal of General Internal Medicine ** | The Inaugural Special Issue for Implementation and Quality Improvement Sciences: A New JGIM Area of Emphasis | Sep 2020 | 2.390 / 161 | DE |
| Ethnicity & Disease ** | Social Determinants of Health and Implementation Research: Three Decades of Progress and a Need for Convergence | Nov 2020 | 1.014 / 61 | US |
| Palgrave Communications * | Expertise in Integration and Implementation for Transformative Research | Dec 2020 | - / - | UK |
| Journal of Health Organization and Management ** | Implementation Science to Practice in Healthcare Organization and Management | Dec 2020 | 1.470 / 35 | UK |
| International Journal of Environmental Research and Public Health * | Air Pollution Interventions: Implementation Research and Data Driven Studies | Jan 2021 | 2.2620 / 78 | CH |
| Health Psychology ** | Reverse Translation: Bridging the Practice-to-Research Gap | ongoing | 3.530 / 148 | US |
| Worldviews on Evidence-Based Nursing ** | Implementing and Sustaining EBP in Real World Healthcare Settings - Worldviews on Evidence-based Nursing | ongoing | 2.650 / 40 | UK |

# Supplementary Table S2. Published implementation science special issues (from year 2000 to March 2020)

| **Journal name** | **Title special issue** | **Volume (issue)** | **Published** | **No. of papers ^c^** | **IF / *h*-index** | **Country** |
| --- | --- | --- | --- | --- | --- | --- |
| Journal of School Psychology ** | Implementation Science in School Psychology | - | in progress | 6 | 3.920 / 83 | UK |
| Frontiers in Public Health; Public Health Policy * | Implementing Public Health Policy Initiatives | - | closed | 2 | 1.680 / 28 | CH |
| Health Policy and Planning ** | Innovations in implementation research in low- and middle-income countries | - | closed | - | 2.717 / 80 | UK |
| Journal of Clinical and Translational Science * | Dissemination and Implementation Sciences in Translational Science | - | closed | - | - / - | UK |
| Administration and Policy in Mental Health and Mental Health Services Research ** | From Research Training to Scientific Advancement: Contributions from the Implementation Research Institute | 47(2) | 2020 | 15 | 2.550 / 58 | DE |
| Frontiers in Public Health -Public Health Policy & Frontiers in Pharmacology - Pharmaceutical Medicine and Outcomes Research * | New Horizons in Health-Promoting: From Methods to Implementation Science | - | 2020 | 2 | 1.680 / 28 | CH |
| International Journal of Environmental Research and Public Health * | Implementation of Interventions in Public Health | 17(4), 1281 | 2020 | 6 | 2.620 / 78 | CH |
| Journal of Community Psychology ** | Applications of translation and implementation science to community psychology | - | 2020 | 13 | 2.120 / 76 | US |

| **Journal name** | **Title special issue** | **Volume (issue)** | **Published** | **No. of papers ^c^** | **IF / *h*-index** | **Country** |
| --- | --- | --- | --- | --- | --- | --- |
| Journal of Nursing Scholarship ** | Special Issue – Journal of Nursing Scholarship: Implementation Science | 52(1), p. 1-123 | 2020 | 13 | 2870 / 72 | UK |
| Psychiatry Research ** | Introduction to Implementation Science: Increasing the Public Health Impact of Research | 283 | 2020 | 13 | 2.370 / 118 | NL |
| AIDS and Behavior ** | Project SOAR: using implementation science to accelerate progress toward achieving the 90-90-90 goals | 23(2), Suppl. | 2019 | 13 | 2.950 / 90 | NL |
| Frontiers in Public Health; Public Health Education and Promotion; Aging and Public Health * | Use of the RE-AIM Framework: Translating Research to Practice with Novel Applications and Emerging Directions | - | 2019 | 15 | 1.680 / 28 | CH |
| Frontiers in Public Health; Translational Medicine; Public Health Education and Promotion * | Methods and Applications in Implementation Science | - | 2019 | 20 | 1.680 / 28 | CH |
| International Journal of Environmental Research and Public Health * | Implementation Research in Chronic Disease Prevention and Control | 16(8), 1403 | 2019 | 6 | 2.620 / 78 | CH |
| Journal of Acquired Immune Deficiency Syndrome (JAIDS) | Implementation Science in the HIV Response: Methodological Challenges and Novel Directions | 82, Suppl. 3 | 2019 | 24 | 3.863 / 142 | US |
| Preventive Medicine ** | Implementation science and population approaches to improve equity in cancer prevention and control | 129, Suppl. | 2019 | 14 | 3.470 / 154 | US |
| Annals of the New York Academy of Sciences ** | Implementation Research and Practice for Early Childhood Development | 1419 | 2018 | 19 | 4.320 / 225 | US |

| **Journal name** | **Title special issue** | **Volume (issue)** | **Published** | **No. of papers ^c^** | **IF / *h*-index** | **Country** |
| --- | --- | --- | --- | --- | --- | --- |
| Behavior Therapy ** | The Intersection of Implementation Science and Behavioral Health | 49(4), p. 477-642 | 2018 | 8 | 3.550 / 97 | US |
| Clinical Psychology: Science and Practice ** | What are we even trying to implement? Considering the relative merits of promoting evidence‐based protocols, principles, practices, or policy | 25(4) | 2018 | 11 | 5.800 / 96 | US |
| Journal of Social Work Education ** | Integrating Evidence-Based Practice and Implementation Science into Academic and Field Curricula | 56, Suppl. 1 | 2018 | 11 | 1.110 / 49 | UK |
| Substance Abuse | Implementation and Quality Improvement: Applying and Advancing Best Practices in Opioid Use Disorder and Addiction Treatment | 39(2) | 2018 | 22 | 2.350 / 39 | US |
| The Journal of the American Board of Family Medicine * | Advancing the Science of Implementation in Primary Health Care | 31(3) | 2018 | 21 | 1960 / 73 | US |
| Pan American Journal of Public Health * | Improving Program Implementation through Embedded Research (iPIER) | 41 | 2017 | 12 | 0.930 / 51 | US |
| Prevention Science ** | Challenges to the Dissemination and Implementation of Evidence-Based Prevention Interventions for Diverse Populations | 18(6) | 2017 | 13 | 2.740 / 76 | NL |
| Research in Social and Administrative Pharmacy ** | Implementation Science | 13(5), p. 889-1036, A1-A8 | 2017 | 18 | 3.130 / 37 | NL |

| **Journal name** | **Title special issue** | **Volume (issue)** | **Published** | **No. of papers ^c^** | **IF / *h*-index** | **Country** |
| --- | --- | --- | --- | --- | --- | --- |
| Administration and Policy in Mental Health and Mental Health Services Research ** | System-Level Implementation of Evidence-Based Practices | 43(6) | 2016 | 14 | 2.550 / 58 | DE |
| Journal of Substance Abuse and Treatment ** | Introduction to the Special Issue on the Studies on the Implementation of Integrated Models of Alcohol, Tobacco, and/or Drug Use Interventions and Medical Care | 60, P1-5 | 2016 | 15 | 2.620 / 93 | NL |
| International Journal of Behavioral Medicine ** | Research to Reality: The Science of Dissemination and Implementation in Behavioral Medicine | 22(3) | 2015 | 16 | 1.990 / 56 | US |
| Journal of Clinical Child & Adolescent Psychology ** | Toward Implementing Physiological Measures in Clinical Child and Adolescent Assessments | 44(2) | 2015 | 11 | 4.240 / 122 | US |
| Maternal and Child Nutrition * | Learning to Effectively Deliver and Promote Adherence in Micronutritient Powder Programs Through Implementation Research | 15, Suppl. 5 | 2015 | 8 | 3.350 / 51 | UK |
| Research on Social Work Practice | Houston Bridging the Research–Practice Gap Symposium | 25(4) | 2015 | 16 | 1.430 / 56 | US |
| Zeitschrift für Evidenz, Fortbildung und Qualität im Gesundheitswesen ** | Das Hohelied der Implementierung wissenschaftlich gesicherter Maßnahmen in die Gesundheitsversorgung [Song of songs about implementation of proven health careinterventions] | 109(2), p. 93-194 | 2015 | 14 | 0.820 / 26 | NL |
| Journal of Clinical Child & Adolescent Psychology ** | Mixed methods and qualitative research in dissemination and implementation science | 43(6) | 2014 | 7 | 4.240 / 122 | US |

| **Journal name** | **Title special issue** | **Volume (issue)** | **Published** | **No. of papers ^c^** | **IF / *h*-index** | **Country** |
| --- | --- | --- | --- | --- | --- | --- |
| Journal of Evidence-Based Social Work ** | Implementation Research | 11(1-2) | 2014 | 19 | - / - | US |
| Administration and Policy in Mental Health and Mental Health Services Research ** | Optimizing Mixed Methods for Implementation Research in Large Systems" and "Regular Papers" | 42(5) | 2013 | 14 | 2.550 / 58 | DE |
| American Journal of Preventive Medicine ** | Implementing Pre-Exposure Prophylaxis (PrEP) in the U.S.: Moving From Evidence to Practice | 44(1), Suppl.2, S59-S172 | 2013 | 22 | 4.435 / 193 | NL |
| Clinical Psychology: Science and Practice | Advances in Applying Treatment Integrity Research for Dissemination and Implementation Science | 20(1), p. 1-126 | 2013 | 10 | 5.800 / 96 | US |
| International Journal of Nursing Studies ** | Implementation Science | 50(4), p. 443-582 | 2013 | 14 | 4.030 / 91 | UK |
| Nursing Research and Practice * | Dissemination and Implementation Research: Intersection between Nursing Science and Health Care Delivery | 2013 | 2013 | 8 | - / - | EG |
| American Journal of Community Psychology ** | Advances in Bridging Research and Practice Using the Interactive System Framework for Dissemination and Implementation | 50(3-4) | 2012 | 24 | 2.120 / 99 | US |
| Depression Research and Treatment * | Implementation Research: Reducing the Research-to-Practice Gap in Depression Treatment | 2012 | 2012 | 11 | 2.170 / 21 | EG |

| **Journal name** | **Title special issue** | **Volume (issue)** | **Published** | **No. of papers ^c^** | **IF / *h*-index** | **Country** |
| --- | --- | --- | --- | --- | --- | --- |
| Administration and Policy in Mental Health and Mental Health Services Research ** | Implementing Evidence-Based Practices | 38(1) | 2011 | 6 | 2.550 / 58 | DE |
| New Directions for Evaluation | Knowledge Utilization, Diffusion, Implementation, Transfer, and Translation: Implications for Evaluation | 2009(124) | 2009 | 8 | 0.220 / 35 | US |
| Research on Social Work Practice ** | - | 19(5) | 2009 | 19 | 1.430 / 56 | US |
| Journal of Public Health Management and Practice ** | Accelerating the Management from Research to Practice | 14(2) | 2008 | 18 | 1.050 / 46 | US |
| American Journal of Preventive Medicine ** | The Dissemination and Utilization of Prevention Research: Increasing Our Knowledge and Understanding | 33(1), Suppl., S1-S80 | 2007 | 7 | 4.435 / 193 | NL |
| Nursing Research and Practice * | Knowledge Translation Research: Advances in Theory and Methods | 56, Suppl. 1 4 | 2007 | 13 | - / - | EG |
| AIDS Education and Prevention | - | 18(4), Suppl. A, 1-2 | 2006 | 16 | 2.040 / 68 | US |
| American Journal of Preventive Medicine ** | Diffusion and Dissemination of Physical Activity Recommendations and Programs to World Populations | 31(4), Suppl. | 2006 | 12 | 4.435 / 193 | NL |
| American Journal of Public Health ** | Diffusion of Innovations | 96(2) | 2006 | 23 | 4.210 / 236 | US |
| Health Psychology ** | Dissemination | 24(5) | 2005 | 11 | 3.530 / 148 | US |
| Journal of Health Communication ** | - | 9, Suppl. 1 | 2004 | 14 | 1.773 / 75 | UK |
| Journal of Community Psychology ** | Bridging the Gap Between Research and Practice in Community Based Substance Abuse Prevention | 28(3), p. 237-373 | 2000 | 10 | 2.120 / 76 | US |

**Reference:**

1. Hodge DR, Lacasse JR. Evaluating Journal Quality: Is the H-Index a Better Measure Than Impact Factors? *Research on Social Work Practice* (2010) 21(2):222-30. doi: 10.1177/1049731510369141.
