# Supplementary figures and images for: Relevant Journals for Identifying Implementation Science Articles: Results of an International Implementation Science Expert Survey
Source: Front Public Health. 2021 Apr 30;9:639192. doi: 10.3389/fpubh.2021.639192 (PMC8119993; doi:10.3389/fpubh.2021.639192)

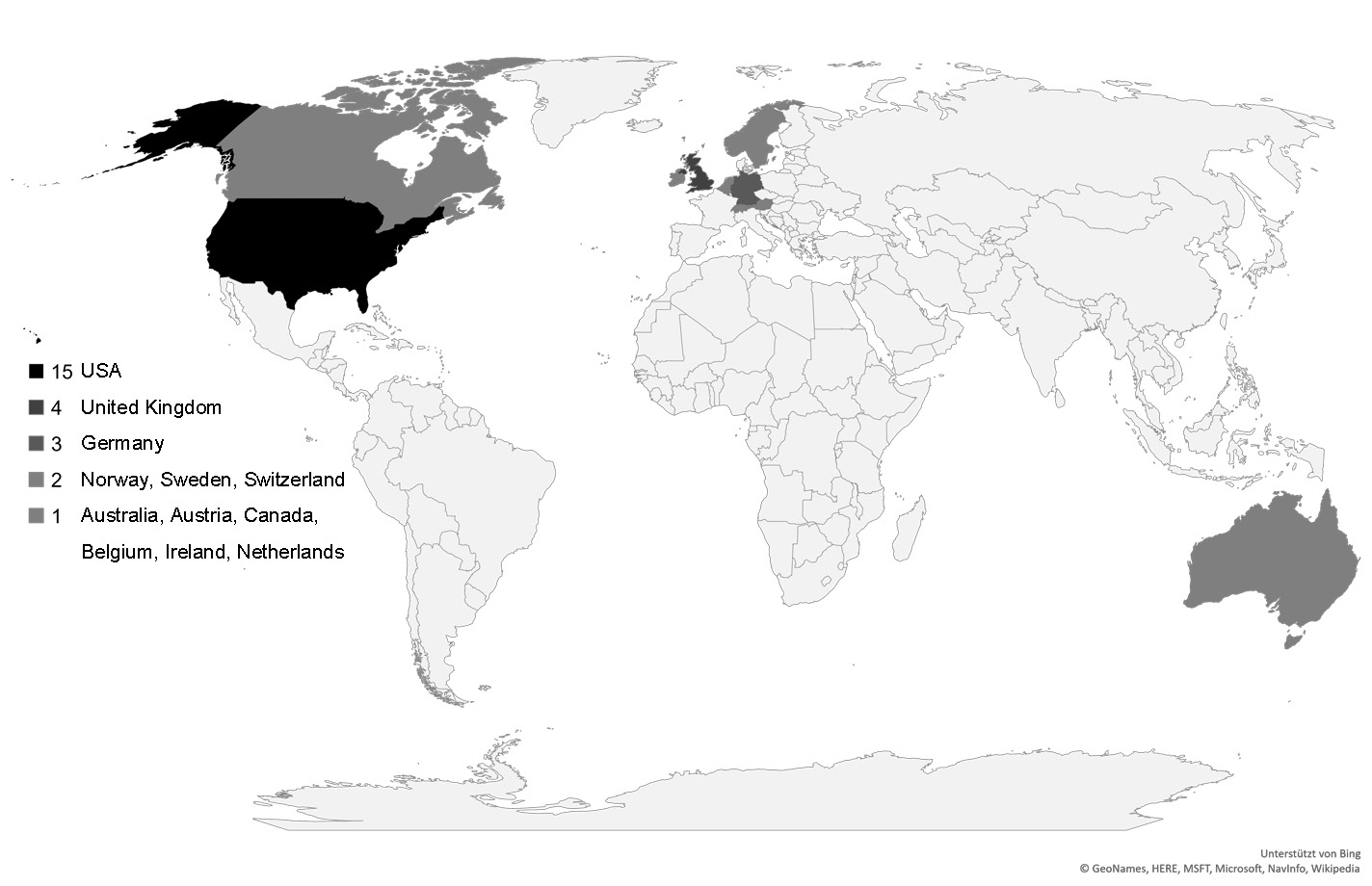

Supplement: Supplementary file 2 [file Image_1.JPEG]
